# Image-based dosimetry for [^225^Ac]Ac-PSMA-I&T therapy and the effect of daughter-specific pharmacokinetics

**DOI:** 10.1007/s00259-024-06681-2

**Published:** 2024-03-21

**Authors:** Grigory Liubchenko, Guido Böning, Mathias Zacherl, Mikhail Rumiantcev, Lena M. Unterrainer, Franz Josef Gildehaus, Matthias Brendel, Sandra Resch, Peter Bartenstein, Sibylle I. Ziegler, Astrid Delker

**Affiliations:** 1 Department of Nuclear Medicine, LMU University Hospital, LMU Munich, Marchioninstrasse 15, 81377 Munich, Germany; 2 Ahmanson Translational Theranostics Division, Department of Molecular and Medical Pharmacology, University of California Los Angeles, Los Angeles, CA USA; 3 SyNergy, University of Munich, Munich, Germany; 4 DZNE - German Center for Neurodegenerative Diseases, Munich, Germany

**Keywords:** PSMA, ^225^Ac, SPECT, Dosimetry, Targeted alpha therapy

## Abstract

**Purpose:**

Although ^221^Fr and ^213^Bi have sufficient gamma emission probabilities, quantitative SPECT after [^225^Ac]Ac-PSMA-I&T therapy remains challenging due to low therapeutic activities. Furthermore, ^221^Fr and ^213^Bi may underlie a different pharmacokinetics due to alpha recoil. We conducted a quantitative SPECT study and a urine analysis to investigate the pharmacokinetics of ^221^Fr and ^213^Bi and the impact on image-based lesion and kidney dosimetry.

**Methods:**

Five patients (7.7 ± 0.2 MBq [^225^Ac]Ac-PSMA-I&T) underwent an abdominal SPECT/CT (1 h) at 24 and 48 h (Siemens Symbia T2, high-energy collimator, 440 keV/218 keV (width 20%), 78 keV (width 50%)). Quantitative SPECT was reconstructed using MAP-EM with attenuation and transmission-dependent scatter corrections and resolution modelling. Time-activity curves for kidneys (CT-based) and lesions (80% isocontour 24 h) were fitted mono-exponentially. Urine samples collected along with each SPECT/CT were measured in a gamma counter until secular equilibrium was reached.

**Results:**

Mean kidney and lesion effective half-lives were as follows: ^213^Bi, 27 ± 6/38 ± 10 h; ^221^Fr, 24 ± 6/38 ± 11 h; 78 keV, 23 ± 7/39 ± 13 h. The ^213^Bi-to-^221^Fr kidney SUV ratio increased by an average of 9% from 24 to 48 h. Urine analysis revealed an increasing ^213^Bi-to-^225^Ac ratio (24 h, 0.98 ± 0.15; 48 h, 1.08 ± 0.09). Mean kidney and lesion absorbed doses were 0.17 ± 0.06 and 0.36 ± 0.1 ${{\text{Sv}}}_{{\text{RBE}}=5}$/MBq using ^221^Fr and ^213^Bi SPECT images, compared to 0.16 ± 0.05/0.18 ± 0.06 and 0.36 ± 0.1/0.38 ± 0.1 ${{\text{Sv}}}_{{\text{RBE}}=5}$/MBq considering either the ^221^Fr or ^213^Bi SPECT.

**Conclusion:**

SPECT/CT imaging and urine analysis showed minor differences of up to 10% in the daughter-specific pharmacokinetics. These variances had a minimal impact on the lesion and kidney dosimetry which remained within 8%.

**Supplementary Information:**

The online version contains supplementary material available at 10.1007/s00259-024-06681-2.

## Introduction

Prostate cancer is the fourth most common malignancy worldwide [1]. [^225^Ac]Ac-PSMA-I&T therapy is a promising alternative for patients with metastatic castration-resistant prostate cancer (mCRPC), who have exhausted all the standard treatments, including prior [^177^Lu]Lu-PSMA-I&T therapy [2–9]. While ^177^Lu emits electrons with a low linear energy transfer (LET) and a range of millimeters in human tissue, ^225^Ac emits alpha particles with a high LET and a range of the order of 100 µm. High-LET radiation shows a higher cytotoxicitiy, resulting in a higher probability for both, tumor control but also side effects. A relative biological effectiveness (RBE) of five, referring to a fivefold higher cytotoxicity, is currently assumed for ^225^Ac compared to ^177^Lu in most dosimetry studies, although even higher RBE values may be conceivable [2, 10–12]. This results in much lower (~ 8 MBq) therapeutic activity used for [^225^Ac]Ac-PSMA-I&T therapy compared to the ~ 7.4 GBq for [^177^Lu]Lu-PSMA-I&T therapy. This, in turn, complicates the post-therapeutic single photon emission computed tomography/computed tomography (SPECT/CT) imaging, although the two ^225^Ac daughter nuclides ^221^Fr (gamma emission probability of 11.4%) and ^213^Bi (gamma emission probability of 25.9%) show sufficient gamma emission probabilities [13]. The lack of a well-established post-treatment imaging procedure obstructs the investigation of the patient-specific pharmacokinetics of ^225^Ac and its subsequent daughters. After the decay of ^225^Ac, in total, four alpha particles are emitted (5.8 MeV, 6.3 MeV, 7.1 MeV, and either 5.9 MeV with 2.2% probability or 8.4 MeV with 97.8% probability) [14]. In principle, these alpha cascades can deliver a high absorbed dose to the lesions; however, the emission of the first alpha particle releases a recoil energy of 100–200 keV, sufficient to break any chemical bond [14–16]. This may cause an additional off-target absorbed dose as unbound daughter nuclides are free to move, unless sufficient internalization and trapping within the tumor cells can be achieved. Thus, the fate of the ^225^Ac daughter nuclides requires careful consideration as they could exacerbate the dose-limiting toxicities [17, 18]. The post-treatment quantitative SPECT may be used to study the pharmacokinetics of the ^225^Ac and subsequent daughters. The second daughter of ^225^Ac, ^221^Fr, has a short half-life (4.8 min) and thus has limited time to migrate away from its parent. Therefore, ^221^Fr is likely to experience a PSMA-driven pharmacokinetics, similar to its mother nuclide. The fourth daughter, ^213^Bi, has a half-life of 45.6 min, which is sufficient to allow for migration away from the ^225^Ac decay site. Therefore, imaging of ^213^Bi could provide insights into the extent of the off-target absorbed dose during [^225^Ac]Ac-PSMA therapy. On the other hand, a very different pharmacokinetics of ^221^Fr and ^213^Bi prevents the signal of both photopeaks from being combined for quantitative SPECT reconstruction to improve the count statistics. So far, patients receiving ^225^Ac-based treatments have often been imaged qualitatively, combining both the 440 and 218 keV peaks or even using three photopeaks by adding the peak around 78 keV [13, 19–22]. So far, published quantitative studies estimated the [^225^Ac]Ac-PSMA lesion and kidney absorbed doses based on knowledge of the [^177^Lu]^177^Lu-PSMA pharmacokinetics that was adjusted for the physical half-life of ^225^Ac [2, 10]. Delker et al. [10] also combined this knowledge with a single ^213^Bi SPECT/CT acquired 24 h post-therapy. However, accounting for a possible daughter migration requires quantitative SPECT imaging of all available photopeaks.

The aim of this study was to perform quantitative SPECT/CT of prostate cancer patients after [^225^Ac]Ac-PSMA-I&T therapy, exploiting all available photopeaks, to compare the pharmacokinetics of the two ^225^Ac daughters ^213^Bi and ^221^Fr. In addition, the ^213^Bi-to-^225^Ac ratio in urine samples was analyzed to provide further validation of the image-based findings. We further compared the quantification of the peak at 78 keV to the results obtained from ^221^Fr and ^213^Bi imaging. Finally, RBE-weighted lesion and kidney absorbed doses were derived, considering also a potentially deviating pharmacokinetics between ^221^Fr and ^213^Bi.

## Methods

### Patients and therapy

This study was a retrospective analysis of the first cycle of five patients diagnosed with mCRPC and treated with 7.7 ± 0.2 MBq [^225^Ac]Ac-PSMA-I&T (Table 1). All patients gave written consent to undergo radioligand therapy (RLT). All data has been irreversibly anonymized before evaluation. The local ethics committee approved the study (project no. 22–0544). Patients were hospitalized for 48 h after injection, as obliged by the German law for radiation protection. Further details on labelling and therapy can be found in the publication by Zacherl et al. [4].

**Table 1:** Patient information

	Injected [^225^Ac]Ac-PSMA-I&T activity (MBq)	PSA before therapy (ng/ml)	Weight (kg)	Age (years)	Analyzed lesions locations*
Patient 1	7.9	6.8	107	66	-
Patient 2	8.0	2356	85	71	LIV
Patient 3	7.8	507	70	68	OSS
Patient 4	7.6	28.9	93	71	LIV
Patient 5	7.4	631	98	80	OSS

*OSS osseous tissue, *LIV* liver; no lesions were located in the SPECT field-of-view for patient 1

### Data acquisition

During their stay in the ward, the patients were scheduled for two post-therapeutic abdominal SPECT/CT scans 24 and 48 h post-injection (p.i.). SPECT/CT acquisition was performed on a dual-headed Symbia T2 SPECT/CT (Siemens Medical Solutions, Erlangen, Germany) equipped with a high-energy collimator and a 3/8″ crystal. Sixteen projections per head were obtained with a matrix size of 128 × 128 pixels (4.7952 × 4.7952 mm^2^) and an acquisition time of 210 s per projection. SPECT/CT imaging used the 440 keV (^213^Bi) and 218 keV (^221^Fr) photopeaks of the ^225^Ac decay chain (width of 20% for both peaks) [10, 13, 18]. In addition, a lower peak at 78 keV (width, 50%) was measured [19–22]. A low-dose CT scan (110 keV, CareDose, slice thickness 3 mm) was performed along with each SPECT scan. To improve patient comfort, patients were positioned with arms down, whereupon the arms were fixed close to the torso via a cloth belt. To obtain additional knowledge about the excretion of ^213^Bi compared to ^225^Ac, two urine samples were collected from all patients right before or right after each SPECT/CT scan.

### Quantitative SPECT reconstruction

SPECT images for all photopeaks were reconstructed using an in-house maximum-a-posterioriexpectation-maximization (MAP-EM) algorithm. Attenuation correction was performed based on the low-dose CT, while scatter correction employed a transmission-dependent scatter correction (TDSC) method [23]. Resolution modelling was based on a pre-simulated 2D point-spread-function model [10]. For the 440 keV peak, the 80th iteration was used for the analysis. For the 78 and 218 keV peaks, the 100th iteration was used for the analysis.

To convert measured counts per second and per voxel (cps/voxel) to becquerel per milliliter (Bq/ml), calibration factors were determined for each energy window. This was performed using a cylindrical calibration phantom (diameter of 25.5 cm; total volume of 8.7 l), homogeneously filled with an activity concentration of 524 Bq/ml (total activity of 4.56 MBq). A large volume-of-interest (VOI) was placed in the reconstructed cylinder volume to extract the average counts per pixel. Imaging and reconstruction of the calibration phantom were carried out using the same protocol as used for patient imaging.

### SUV analysis and dosimetry

Reconstructed SPECT/CT images were analyzed using PMOD (Version 3.609, PMOD Technologies, Zurich, Switzerland). For each of the patients, the kidneys and all of the lesions that were visible in the abdominal region were evaluated (Table 1; no lesions were available in the field of view for patient 1). Before segmentation, all SPECT images were filtered for noise suppression using a Gaussian filter with a full-width-half-maximum of 30 mm. This filter parameter was chosen as it provides the best compromise between signal-to-noise ratio and recovery coefficients in kidneys and lesions, similar to a previous study [10]. Both kidneys were segmented using the CT accompanying each SPECT scan. Lesions were segmented using an isocontour of 80% of maximum tissue intensity on the SPECT acquired at 24 h post-therapy. These VOIs were transferred to the SPECT at 48 h afterwards. In case of misalignment with the SPECT/CT 24 h p.i., VOIs were manually shifted. Lesion segmentation was performed separately for each of the peaks.

Mean standardized uptake values (SUV) in the VOIs were calculated for kidneys and lesions at 24 and 48 h p.i. for the three peaks. The ^213^Bi and ^221^Fr SUV were tested for correlation (via Python 3.9 Pearson correlation coefficients). These SUVs were then also compared to the SUV for 78 keV.

The total VOI activities at 24 and 48 h p.i. were then loaded into Spyder (Python 3.9) software, and a conventional mono-exponential model (Eq. 1) was fitted to the data points to generate the time-activity curve (TAC). The mono-exponential model is given by the following:

1 $$A\left(t\right)=A\left(t_0\right)\cdot e^{\left(-\frac{\text{ln}\left(2\right)}{T_{1/2}}\cdot t\right)},$$

where ${T}_{1/2}$ is the effective half-life (${T}_{1/2}=\frac{{\text{ln}}\left(2\right)}{{\lambda }_{{\text{bio}}}+{\lambda }_{{\text{phys}}}}$, where ${\lambda }_{{\text{bio}}}$ and ${\lambda }_{{\text{phys}}}$ are biological and physical decay constants, respectively); $A(t)$ is the total VOI activity at a given time point $t$ post-injection. The effective half-life (${T}_{1/2}$) and initial activity ($A\left({t}_{0}\right)$) were used as the free parameters during the fitting process.

From the fitted model (Eq. 1), the lesion and kidney effective half-lives for ^213^Bi and ^221^Fr were determined from the 440 and 218 keV SPECT images, respectively. These effective half-lives were tested for correlation afterwards (via Python 3.9 Pearson correlation coefficients). The lesion and kidney effective half-lives were also derived based on imaging of the 78 keV, and respective values were compared with those obtained for ^213^Bi and ^221^Fr. The RBE-weighted absorbed doses of the kidneys and lesions were estimated based on the MIRD formalism [24] with the usage of a RBE of 5 [11]. Kidney and lesion dosimetry accounted for self-irradiation only. The RBE-weighted absorbed doses were calculated using three different methods. For the first two methods, the 440 (^213^Bi) and 218 (^221^Fr) keV peaks were used separately. Here, it was assumed that the localization of ^225^Ac and all subsequent daughters is described by SPECT imaging of either 440 (^213^Bi) (referred to as method 1 in this paper) or 218 (^221^Fr) keV peak (referred to as method 2 in this paper), respectively. For the third method (referred to as method 3 in this paper), the RBE-weighted absorbed doses for ^225^Ac were derived by combining the ^213^Bi and ^221^Fr components. The first component is based on the assumption that the localization of ^225^Ac, ^221^Fr, and ^217^At is described by SPECT imaging of the ^221^Fr photopeak at 218 keV due to the short half-lives of ^221^Fr and ^217^At (4.8 min and 32 ms, respectively [13]). More precisely, dosimetry was performed based on the effective half-life as extracted from ^221^Fr SPECT imaging at 24 and 48 h p.i. and by summing up the energy deposition due to self-irradiation by ^225^Ac,^221^Fr, and ^217^At. For the second component, it was similarly assumed that ^213^Bi SPECT imaging is representative for ^213^Bi and all subsequent daughters. S-values were taken from the open-access online resource OpenDose [25]. The SPECT images of the 78 keV peak were not included in the dosimetry analysis.

Additionally, the statistical analysis via Python 3.9 Wilcoxon signed-rank testing was performed to compare the kidney and lesion absorbed doses. For every patient, each kidney was compared with each lesion (a total of 13 kidney-lesion pairs). This method was chosen to provide sufficient data for comparison and to increase statistical power.

### Urine analysis

For each of the urine samples, 1 ml was pipetted into a test tube immediately after sample collection and analyzed for at least 6 h in a HIDEX gamma counter (HIDEX, Turku, Finland), until secular equilibrium was reached. Counts were measured in the same photopeaks as for the SPECT acquisitions. For ^213^Bi, the obtained data points were then fitted by using a model (Eq. 2), which describes the total activity of ^213^Bi via two components: ^213^Bi, which is generated by ^225^Ac decaying in the samples (first term of the equation), and ^213^Bi activity being already present in the samples at the time of sample collection (second term of the equation):

2 $${A}_{{\text{Bi}}}\left(t\right)={A}_{{\text{Ac}}}\left(0\right)\left(\frac{{\lambda }_{{\text{Bi}}}}{{\lambda }_{{\text{Bi}}}-{\lambda }_{{\text{Ac}}}}\right)\left({e}^{{-\lambda }_{{\text{Ac}}}\cdot t}-{e}^{-{\lambda }_{{\text{Bi}}}\cdot t}\right)+{A}_{{\text{Bi}}}\left(0\right){e}^{-{\lambda }_{{\text{Bi}}}\cdot t}.$$

$t$ is the time passed after sample collection; ${A}_{{\text{Bi}}}\left(t\right)$ is the total ^213^Bi activity over time; ${A}_{{\text{Ac}}}\left(0\right)$ and ${A}_{{\text{Bi}}}\left(0\right)$ refer to the ^225^Ac and ^213^Bi activity at the time of sample collection (*t* = 0), and ${\lambda }_{{\text{Bi}}}$ and ${\lambda }_{{\text{Ac}}}$ are the ^213^Bi and ^225^Ac physical decay constants, respectively [26]. In this equation, the short-lived daughter nuclides ^221^Fr and ^217^At are ignored, assuming that ^225^Ac decays directly into ^213^Bi. By fitting the aforementioned model to the data points acquired by the gamma counter measurement, ${A}_{{\text{Ac}}}\left(0\right)$ and ${A}_{{\text{Bi}}}\left(0\right)$ can be derived.

## Results

### Quantitative SPECT images

**Fig. 1:**
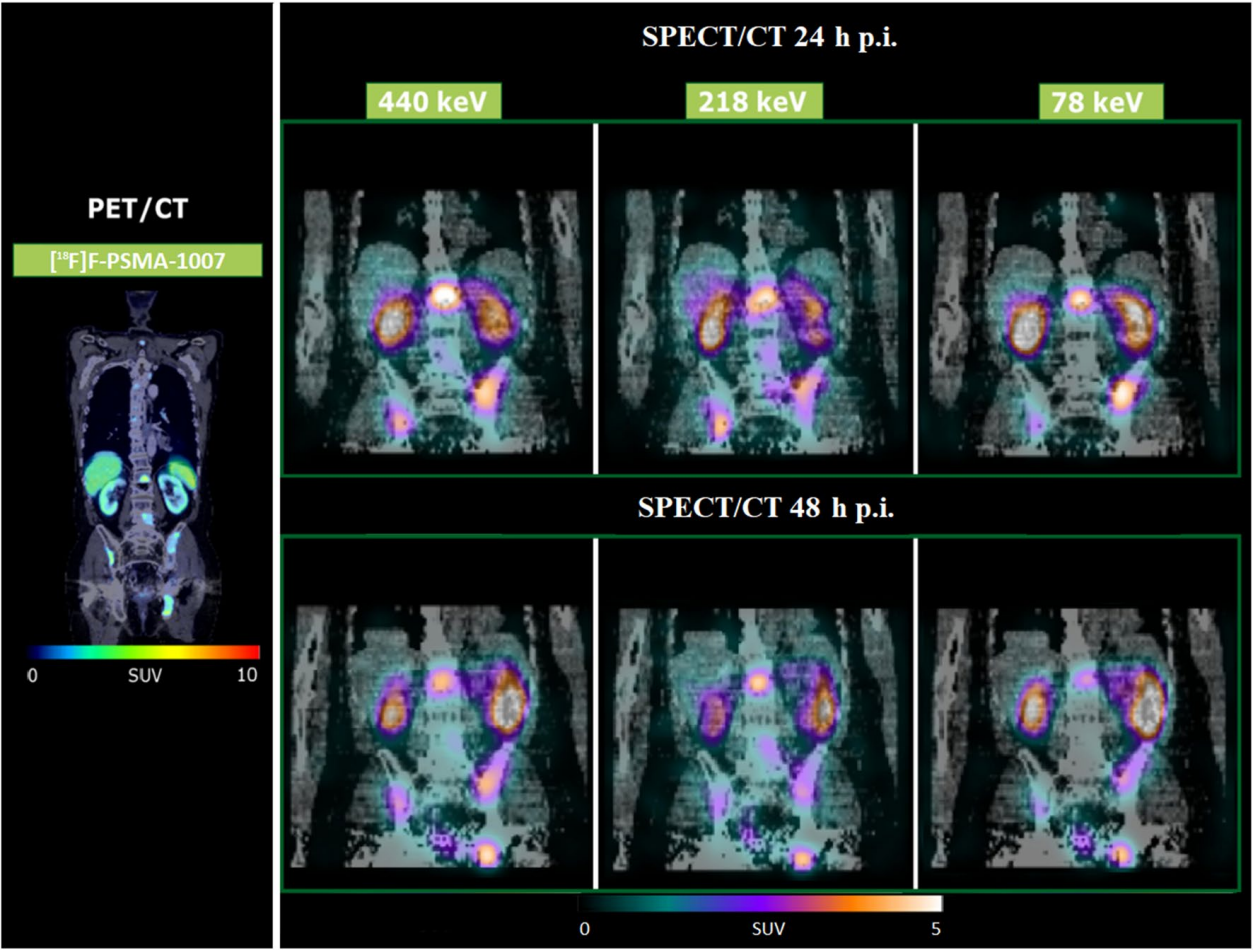
Pre-therapy PET/CT and post-filtered SPECT/CT acquired at 24 and 48 h post-treatment (Gaussian post-filter, FWHM 30 mm) for patient 3

Figure 1 shows the post-filtered SPECT/CT images exemplarily for patient 3. In addition, the PET/CT performed 2 weeks prior to treatment is shown as the diagnostic gold standard.

### Kidney and lesion SUV

**Fig. 2:**
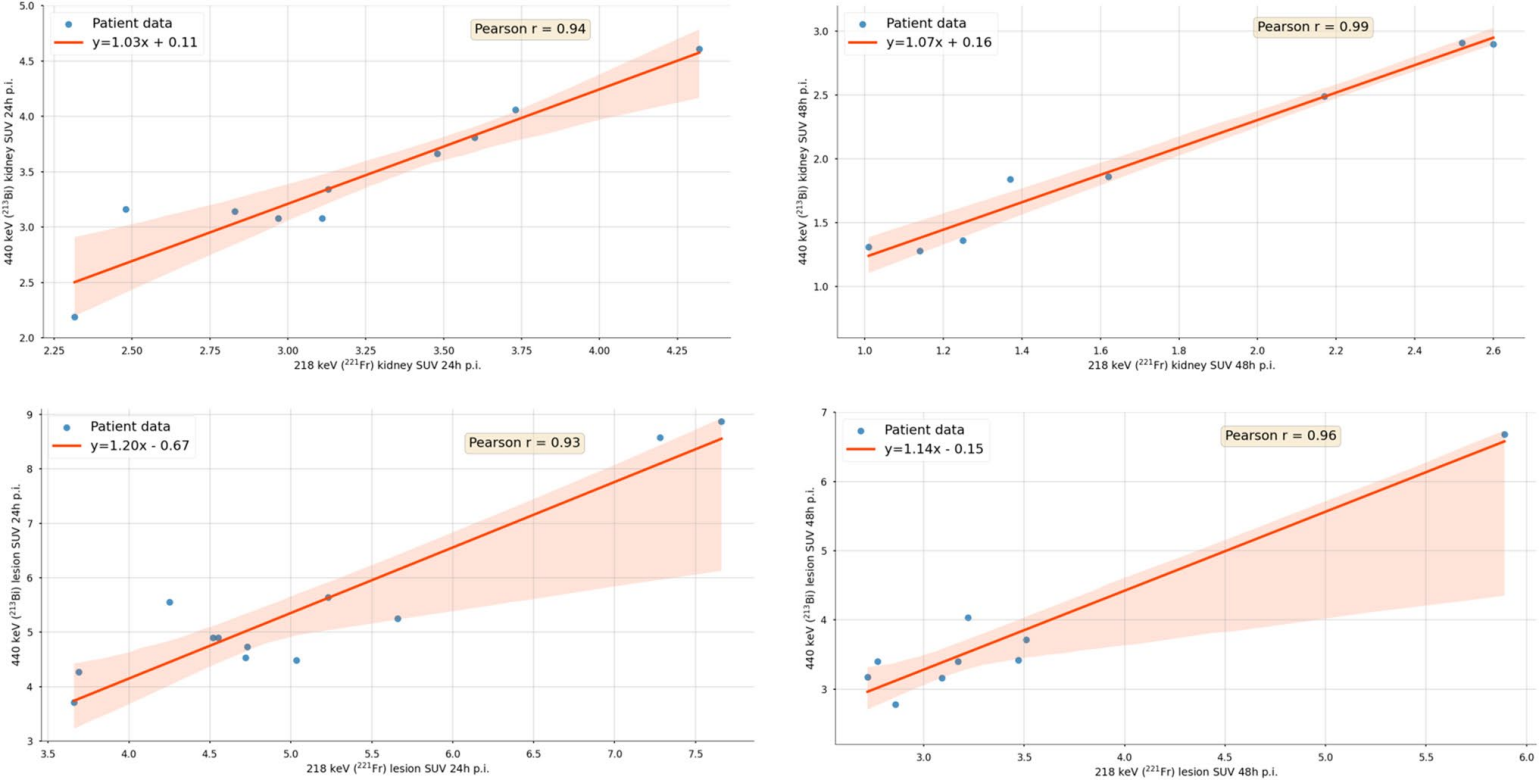
SUV for kidneys (top) and lesions (bottom) for 440 keV (^213^Bi) and 218 keV (^221^Fr) at 24 (left) and 48 h (right) post-treatment. All subplots include 95% confidence intervals and Pearson correlation coefficients. SUVs are not recovery-corrected

**Fig. 3:**
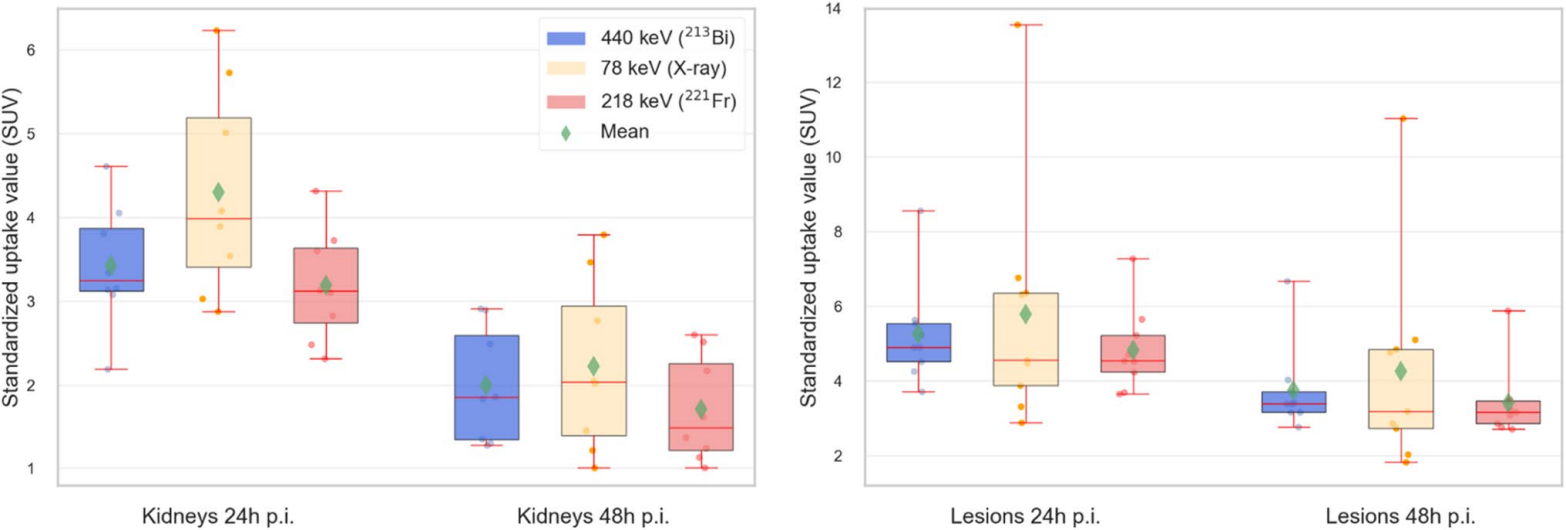
SUV for kidneys (*n* = 8) (left) and lesions (*n* = 9) (right) for 440 keV (^213^Bi), 218 keV (^221^Fr), and 78 keV. Values are not recovery-corrected

In Fig. 2, the correlation plots for the SUV for 440 keV (^213^Bi) and 218 keV (^221^Fr) at 24 and 48 h p.i. are shown for a total of eight kidneys and nine lesions. The left kidney of patients 3 and 5 were excluded from the analysis due to high intestinal uptake at 48 h p.i., which superimposed onto the left kidney. The SUVs show a very strong correlation for both time points and in both, the lesions (24 h, *r* = 0.93, *p*-value = 0.0006; 48 h, *r* = 0.96, *p*-value < 0.0001) and the kidneys (24 h, *r* = 0.94, *p*-value = 0.0004; 48 h, *r* = 0.99, *p*-value < 0.0001). Figure 3 shows the kidney and lesion SUV for all three peaks (440 keV, 218 keV, and 78 keV) at 24 and 48 h post-treatment. Further details on the SUV per energy window and time point are found in Table 2. In addition, Table 3 shows the corresponding SUV ratios at 24 and 48 h post-treatment.

**Table 2:** Lesion and kidney SUVs, effective half-lives, and segmented volumes for all energy windows (mean values ± standard deviation). Values are not recovery-corrected

Energy	Compartment	SUV 24 h p.i	SUV 48 h p.i	Segmented volume (ml)	Effective half-life (hours)
218 keV (^221^Fr)	Lesions	4.8 ± 1.2	3.4 ± 0.9	23 ± 10	38 ± 11
	Kidneys	3.2 ± 0.6	1.7 ± 0.6	247 ± 81	24 ± 6
440 keV (^213^Bi)	Lesions	5.3 ± 1.6	3.8 ± 1.1	23 ± 7	38 ± 10
	Kidneys	3.4 ± 0.6	2.0 ± 0.6	247 ± 81	27 ± 6
78 keV (X-rays)	Lesions	5.8 ± 3.1	4.3 ± 2.7	27 ± 18	39 ± 13
	Kidneys	4.3 ± 1.1	2.2 ± 1.0	247 ± 81	23 ± 7

**Table 3:** SUV ratios (mean ± standard deviation) for all peaks at 24 and 48 h post-treatment. Values are not recovery-corrected. The table includes the percentage change (mean values ± standard deviation) between 48 and 24 h

SUV ratio	Kidneys			Lesions		
	24 h	48 h	% change 48 to 24 h	24 h	48 h	% change 48 to 24 h
440 to 218 keV	1.08 ± 0.1	1.18 ± 0.1	9 ± 8	1.09 ± 0.1	1.10 ± 0.1	2 ± 15
440 to 78 keV	0.81 ± 0.1	0.95 ± 0.1	15 ± 12	1.03 ± 0.3	1.06 ± 0.4	2 ± 13
218 to 78 keV	0.76 ± 0.1	0.81 ± 0.1	6 ± 10	0.95 ± 0.3	0.97 ± 0.4	1 ± 17

### Effective half-lives of ^213^Bi and ^221^Fr

**Fig. 4:**
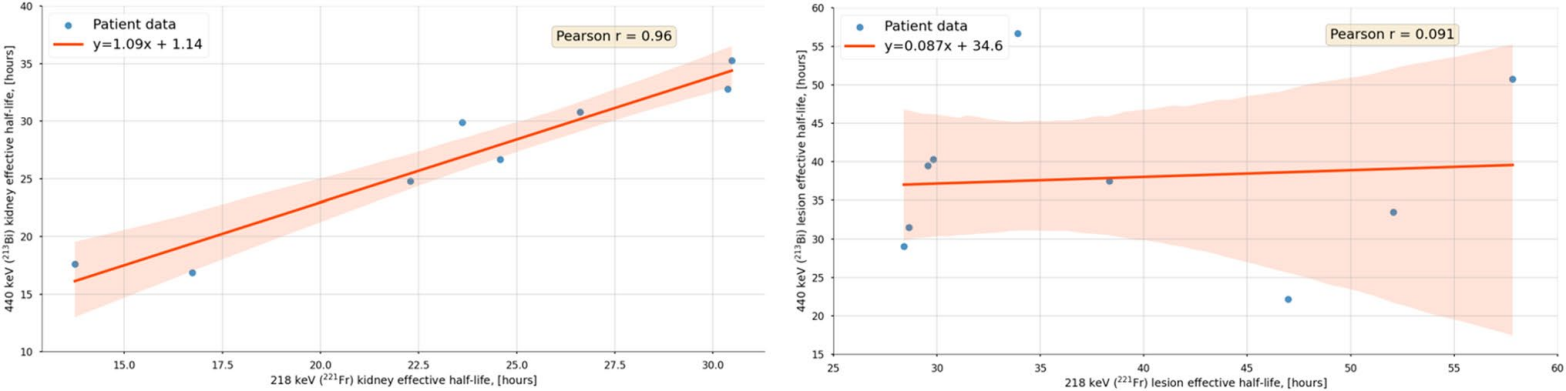
Correlations plots for kidney (left) and lesion (right) effective half-lives for ^213^Bi and ^221^Fr. Both subplots include 95% confidence intervals and Pearson correlation coefficients

**Fig. 5:**
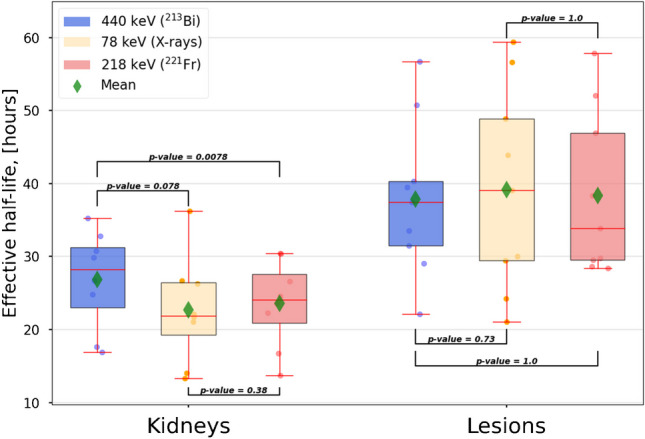
Effective half-lives for kidneys (*n* = 8) and lesions (*n* = 9) for 440 keV (^213^Bi), 218 keV (^221^Fr), and 78 keV. *p*-values for the statistical analysis via the Wilcoxon signed-rank test are added

In Fig. 4, the correlation plots for the kidney (*n* = 8) and lesion (*n* = 9) effective half-lives for 440 keV (^213^Bi) and 218 keV (^221^Fr) are shown. No correlation (*r* = 0.091, *p*-value = 0.82) was found for the lesions, while a very strong correlation (*r* = 0.96, *p*-value = 0.00012) was found for the kidneys. It is worth mentioning that for all kidneys, ^221^Fr showed a shorter effective half-life than ^213^Bi. The statistical analysis via the Wilcoxon signed-rank test showed no statistical difference (*p*-value = 0.69) for the ^221^Fr and ^213^Bi lesion effective half-life. For the kidneys, however, the Wilcoxon signed-rank test showed a significant difference (*p*-value = 0.0078) between ^221^Fr and ^213^Bi effective half-lives. Figure 5 visualizes the effective half-lives for both, the kidneys and the lesions for 440 keV, 218 keV, and 78 keV. Further details are found in Table 2.

### Kidney and lesion dosimetry

**Fig. 6:**
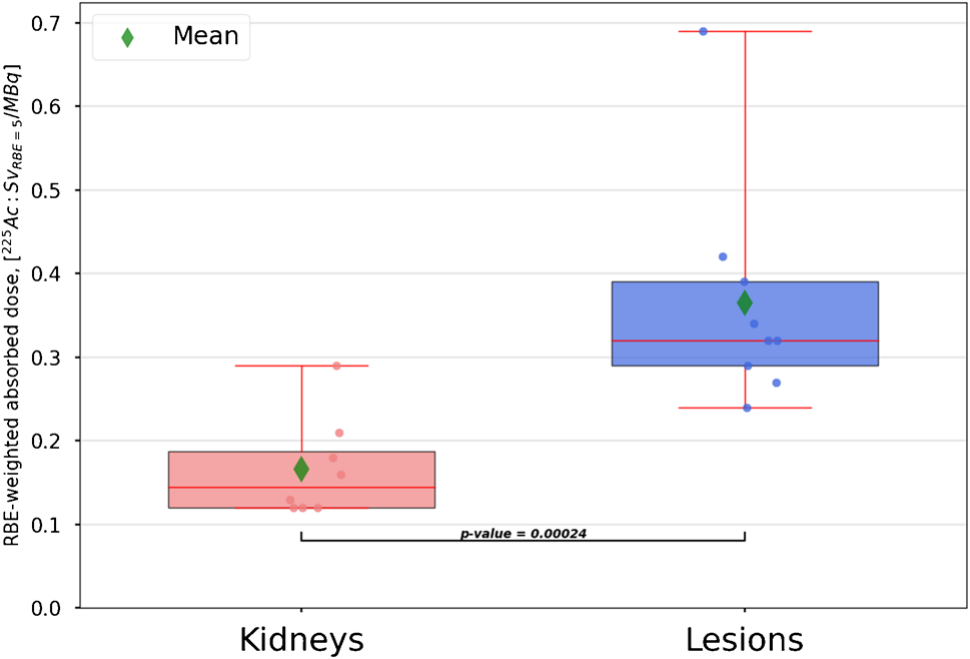
RBE-weighted absorbed doses for kidneys (*n* = 8) and lesions (*n* = 9) for ^225^Ac. A *p*-value for the statistical analysis via the Wilcoxon signed-rank test is also included. Values are not recovery-corrected

In Fig. 6, the kidney and lesion RBE-weighted absorbed doses per injected activity of [^225^Ac]Ac-PSMA-I&T are presented. The mean RBE-weighted kidney and lesion absorbed doses were 0.17 ± 0.06 and 0.36 ± 0.1 ${{\text{Sv}}}_{{\text{RBE}}=5}$/MBq, respectively. Even with consideration of a potential daughter mobility, statistical analysis revealed significantly higher RBE-weighted absorbed doses to the lesions compared to the RBE-weighted kidney absorbed dose (*p* = 0.00024). In Table 4, the RBE-weighted lesion and kidney absorbed doses are shown, considering either the daughter-specific pharmacokinetics (method 3) or either the ^221^Fr or the ^213^Bi SPECT only (methods 1 and 2).

**Table 4:** RBE-weighted kidney and lesion absorbed doses (mean values ± standard deviation), considering either the daughter-specific pharmacokinetics (method 3) or either the ^221^Fr or the ^213^Bi SPECT only (methods 1 and 2). The table includes the percentage differences (mean values ± standard deviation) between the three methods. Values are not recovery-corrected

Compartment	RBE-weighted absorbed dose, [${{\text{Sv}}}_{{\text{RBE}}=5}$/MBq]			% difference		
	Method 1 (^213^Bi)	Method 2 (^221^Fr)	Method 3 (^221^Fr + ^213^Bi)	Method 1 vs. method 2	Method 1 vs. method 3	Method 2 vs. method 3
Kidneys	0.18 ± 0.06	0.16 ± 0.05	0.17 ± 0.06	12 ± 4	8 ± 2	4 ± 2
Lesions	0.38 ± 0.1	0.36 ± 0.1	0.36 ± 0.1	6 ± 16	4 ± 11	2 ± 5

### Urine data

**Fig. 7:**
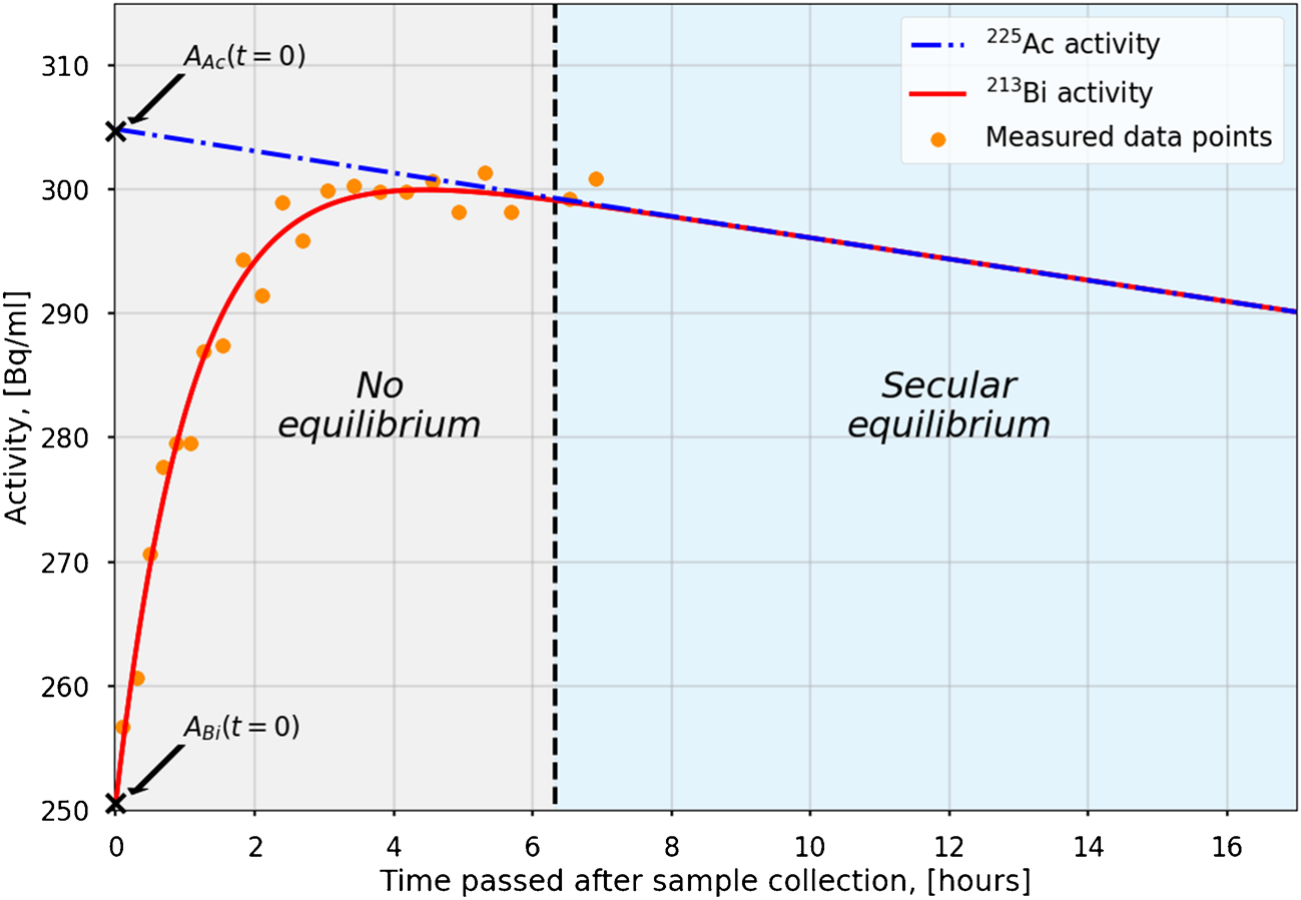
Continuous gamma counter measurement of a urine sample collected from patient 4 approximately 24 h post-treatment. All measurements (orange dots) were fitted using Eq. 3. 6.3 h (black dashed line) after sample collection, secular equilibrium between ^213^Bi and ^225^Ac was reached [27]

Figure 7 shows an example of a continuous urine sample measurement (24 h p.i., patient 4). The y-intercepts of the ^213^Bi and ^225^Ac activity curves are used to determine the ^213^Bi and ^225^Ac urine activity concentrations at the time of sample collection.

Table 5 summarizes the ^213^Bi and ^225^Ac urine activity concentrations at approximately 24 and 48 h post-injection. The mean ratio of ^213^Bi-to-^225^Ac activities in the urine increased from 0.98 ± 0.15 at 24 h to 1.08 ± 0.09 48 h post-treatment. This indicates an increase of (10 ± 9)% of ^213^Bi compared to ^225^Ac activity. The observed increase of the ^213^Bi-to-^221^Fr SUV ratio from 24 to 48 h post-injection was found to be (9 ± 8)% (Table 3).

**Table 5:** Urine activity concentrations per injected activity for ^225^Ac and ^213^Bi at 24 and 48 h post-injection. The ratios of ^213^Bi-to-^225^Ac are also included in the table

	Time post-injection (hours)	^225^Ac urine activity concentration (Bq/ml)	^213^Bi urine activity concentration (Bq/ml)	Ratio, ^213^Bi/^225^Ac
Patient 1	17.0	242.6	218.9	0.90
	42.5	38.7	41.7	1.08
Patient 2	18.0	232.9	248.3	1.07
	41.3	38.8	40.3	1.04
Patient 3	22.0	61.1	76.0	1.24
	39.0	44.1	55.7	1.26
Patient 4	17.0	303.9	250.6	0.82
	40.6	42.9	43.3	1.01
Patient 5	18.6	128.6	113.3	0.88
	42.0	113.2	115.6	1.02

## Discussion

This work aimed to perform quantitative post-treatment SPECT/CT of prostate cancer patients, who have undergone [^225^Ac]Ac-PSMA-I&T therapy, to study and compare the pharmacokinetics of the two SPECT-imageable daughter nuclides ^221^Fr and ^213^Bi. Overall, the data suggests that ^213^Bi, which was found to be accumulating in the urine, shows a longer effective half-life in the kidneys compared to ^221^Fr and might experience a non-PSMA-driven behavior. Quantitative SPECT imaging for ^213^Bi and ^221^Fr further served as a basis for patient-specific dosimetry.

Very strong correlations were observed for the ^213^Bi and ^221^Fr kidney effective half-lives and SUVs. The same holds for the lesion SUV. The Wilcoxon signed-rank test showed statistically significant differences for the ^213^Bi and ^221^Fr kidney effective half-lives, while none was observed for the lesion effective half-lives. For the kidneys, this suggests that although the SUVs and effective half-lives for the kidneys are closely correlated for the ^213^Bi and ^221^Fr, the absolute rate of excretion of these radionuclides from the patient may differ. This could occur due to the release of unbound and non-internalized ^213^Bi from the tumor sites, whereupon free ^213^Bi is known to accumulate in the kidneys [15, 17]. For four out of five patients, the ratio of ^213^Bi-to-^225^Ac in the urine clearly increased from 24 up to 48 h post-treatment, which supports the findings from image analysis that unbound ^213^Bi is present with a non-PSMA driven behavior. Moreover, the average ratio of ^213^Bi-to-^225^Ac in the urine increases by about (10 ± 9)% between 24 and 48 h p.i., which is comparable to the (9 ± 8)% increase in the kidney SUV ratio between ^213^Bi and ^221^Fr in the same time period as obtained from the SPECT analysis. Therefore, while ^221^Fr and ^217^At may be considered PSMA-driven (due to their short half-lives), this assumption is of limited validity for unbound ^213^Bi and subsequent daughters. Additional validation would be desirable, for example with the help of biokinetic modelling. Furthermore, only five patients were analyzed in this study, which provides limited statistical power, and therefore, further patient data should be collected and analyzed to confirm the results presented in this paper. However, these findings are consistent with the study by Kruijff et al. [15], who injected [^225^Ac]Ac-polymersomes into mice and found that the kidneys were the primary organ accumulating unbound ^213^Bi to a similar order of magnitude.

Although no correlation was found for the lesion effective half-lives, the lesion SUVs are very strongly correlated for ^213^Bi and ^221^Fr. However, comparing quantitative image results for lesions is particularly challenging, as both, the absolute quantitative error and its associated uncertainty, are known to increase for smaller structures. In this study, we omitted any partial volume effect and recovery corrections, as they are usually based on idealized phantom measurements, neglecting the object-specific conditions such as object shape and foreground-to-background ratio. However, e.g., the potential of AI-based improvement of image resolution could be investigated in future studies [28].

Assessing the mobility of decay daughters via patient imaging is useful for patient-specific dosimetry. With consideration of a daughter mobility (Fig. 6 and Table 4 (method 3)), the RBE-weighted lesion absorbed doses were found to be still significantly higher compared to RBE-weighted kidney absorbed doses, with means of 0.36 and 0.17 ${{\text{Sv}}}_{{\text{RBE}}=5}$/MBq, respectively. This difference between the lesion and kidney RBE-weighted absorbed doses may even be larger, considering that the recovery coefficients for the lesions are generally smaller than for the kidneys due to the smaller size of the lesions (Table 2) [10]. Using methods 1 and 2 based on either the ^221^Fr or the ^213^Bi SPECT only provided similar results to method 3 (differences within 8%; Table 4). Thus, although there are observable differences in the daughter-specific pharmacokinetics, these differences and the impact on patient-specific dosimetry were small for the patient cohort in this study. These findings may leave room for combining the signal from all available energy windows during reconstruction to improve the SPECT count statistics.

It should be mentioned that post-therapeutic imaging was only performed until 48 h post-injections as patients were discharged afterwards. At least three imaging time points would be desirable for a mono-exponential fit function as well as the lesion and kidney dosimetry. However, this option was not available due to the early discharge and the long acquisition time of 1 h that is still needed for ^225^Ac SPECT imaging. Further research should focus on a reduction of acquisition times in the scope of targeted alpha therapy. This could be achieved by improving both, hardware and software technology. For instance, thicker crystals are highly desirable particularly in the context of imaging of alpha emitters and low therapeutic activities. Further, AI-based techniques as proposed by Leube et al. [29] and Ryden et al. [30] could assist in a further reduction of scanning time. Finally, an RBE of 5 was used for the absorbed dose calculations. A simulation study by Rumiantcev et al. [12] has shown that the RBE may be dependent on the absorbed dose, i.e., the RBE of [^225^Ac]Ac-PSMA-I&T compared to [^177^Lu]Lu-PSMA-I&T is decreasing with increasing absorbed dose. These findings have to be further exploited in in vitro and in vivo experiments.

In a study carried out by Kratochwil et al. [2], dosimetric estimations for 14 prostate cancer patients injected with [^225^Ac]Ac-PSMA-617 were performed based on TACs derived from [^177^Lu]Lu-PSMA-617 scans that were adjusted for the physical half-life of ^225^Ac. They estimated the RBE-weighted kidney absorbed dose to be 0.7 ${{\text{Sv}}}_{{\text{RBE}}=5}$/MBq. This estimate is about four times larger compared to the findings in this study. This difference could arise due to the variations in the patient cohorts, but also due to the differences in imaging and dosimetry methodology. Noise, post-filtering, and the presence of high-energy photons impair image quality and quantification for ^225^Ac SPECT imaging. Thus, the higher kidney absorbed doses as observed by Kratochwil et al. [2] may be partially attributed to the comparatively better quantification of ^177^Lu SPECT images. A study performed by Delker et al. [10] investigated eight prostate cancer patients undergoing combined [^177^Lu]Lu-PSMA-I&T/[^225^Ac]Ac-PSMA-I&T therapy. Similar to the study by Kratochwil et al. [2], [^225^Ac]Ac-PSMA-I&T effective half-lives were extrapolated from the patient-specific [^177^Lu]Lu-PSMA-I&T serial SPECT imaging. These ^177^Lu-based half-lives were then combined with a single post-filtered ^213^Bi SPECT/CT 24 h post-treatment. This approach resulted in effective half-lives of 33 ± 19 and 61 ± 55 h for [^225^Ac]Ac-PSMA-I&T in kidneys and lesions, respectively, as well as RBE-weighted absorbed doses of 0.28 ± 0.14 and 0.22 ± 0.21 ${{\text{Sv}}}_{{\text{RBE}}=5}$/MBq. Considering a reported ^177^Lu-to-^225^Ac recovery ratio of 2.4 (spherical volume of 200 ml), the kidney dosimetry by Delker et al. [10] and Kratochwil et al. [2] may be regarded as comparable. The RBE-weighted kidney absorbed doses in this work are slightly lower compared to the findings by Delker et al. [10], although both studies include SPECT imaging of the ^225^Ac daughters. However, in this study, the attempt was to measure effective half-lives based on sequential ^225^Ac SPECT imaging, instead of using sequential ^177^Lu imaging as a surrogate. This sequential ^225^Ac SPECT imaging approach yielded a lower kidney effective half-life (33 ± 19 h vs. 27 ± 6 h), and thus lower RBE-weighted absorbed dose.

The 78 keV peak has shown comparable quantification characteristics compared to the 440 (^213^Bi) and 218 (^221^Fr) keV peaks, providing not only valuable qualitative but also quantitative information. Similar to a study by Benabdallah et al. [21], we used a relatively wide energy window for the low-energy peak of the ^225^Ac spectrum. Such a wide energy spectrum especially at low energies is likely to increase the fraction of scattered counts in the peak energy window, implying the need for robust scatter correction. Due to the latter reason and because window-based scatter correction is particularly challenging in the extreme low-count regime, we decided to use a transmission-dependent scatter correction as proposed by Sohlberg et al. [23], which estimates the number of scattered photons per iteration based on the current image estimate and the patient’s CT.

No statistical difference was observed for the lesion and kidney effective half-lives between neither 78 keV and 440 keV (^213^Bi) nor 78 keV and 218 keV (^221^Fr) peaks. The change in SUV ratios between 218 (^221^Fr) and 78 keV remains within 6% between 24 and 48 h p.i. for lesions and kidneys. The same holds for lesions for 440 (^213^Bi) and 78 keV. In future studies, the peak at 78 keV could be used to improve the count statistics during reconstruction. Usmani et al. [19], Rasheed et al. [20], and Vatsa et al. [22] already investigated a similar reconstruction approach for [^225^Ac]Ac-PSMA treatment, so far mainly aiming at a qualitative improvement of post-therapeutic images. However, the results from this analysis suggest also an extension of multi-peak reconstruction for image quantification and dosimetry.

## Conclusion

A pharmacokinetic and dosimetry study for [^225^Ac]Ac-PSMA-I&T therapy was performed for the two imageable daughters of ^225^Ac, ^213^Bi, and ^221^Fr. The kidney SUV ratio of ^213^Bi compared to ^221^Fr was found to increase by on average (9 ± 8)% from 24 to 48 h p.i., while the corresponding ^213^Bi-to-^225^Ac ratio of urine activities increased by (10 ± 9)%. In addition, ^221^Fr and ^213^Bi kidney effective half-lives were significantly different. However, the use of the ^213^Bi or ^221^Fr post-therapeutic scans resulted in comparable (within 8%) absorbed dose estimates for the kidneys and lesions, compared to a dosimetry approach taking into account the daughter-specific pharmacokinetics. This finding may offer the opportunity to simultaneously image both photopeaks for dosimetry purposes to improve the counting statistics. Quantitative results for the peak at 78 keV are comparable to those obtained for the photopeaks of ^213^Bi and ^221^Fr, suggesting that this signal could also be used to improve the signal strength for quantitative ^225^Ac SPECT imaging.

### Supplementary Information

Below is the link to the electronic supplementary material.

Supplementary file1 (DOCX 261 KB)

## Data Availability

Please contact the corresponding author.
