# Supplementary material for: Image-based dosimetry for [225Ac]Ac-PSMA-I&T therapy and the effect of daughter-specific pharmacokinetics
Source: Eur J Nucl Med Mol Imaging. 2024 Mar 21;51(8):2504–14. doi: 10.1007/s00259-024-06681-2 (PMC11178588; doi:10.1007/s00259-024-06681-2)
Supplement: Supplementary file 1 — Supplementary file1 (DOCX 261 KB) [file 259_2024_6681_MOESM1_ESM.docx]

*Supplement*

**Time-activity curves**


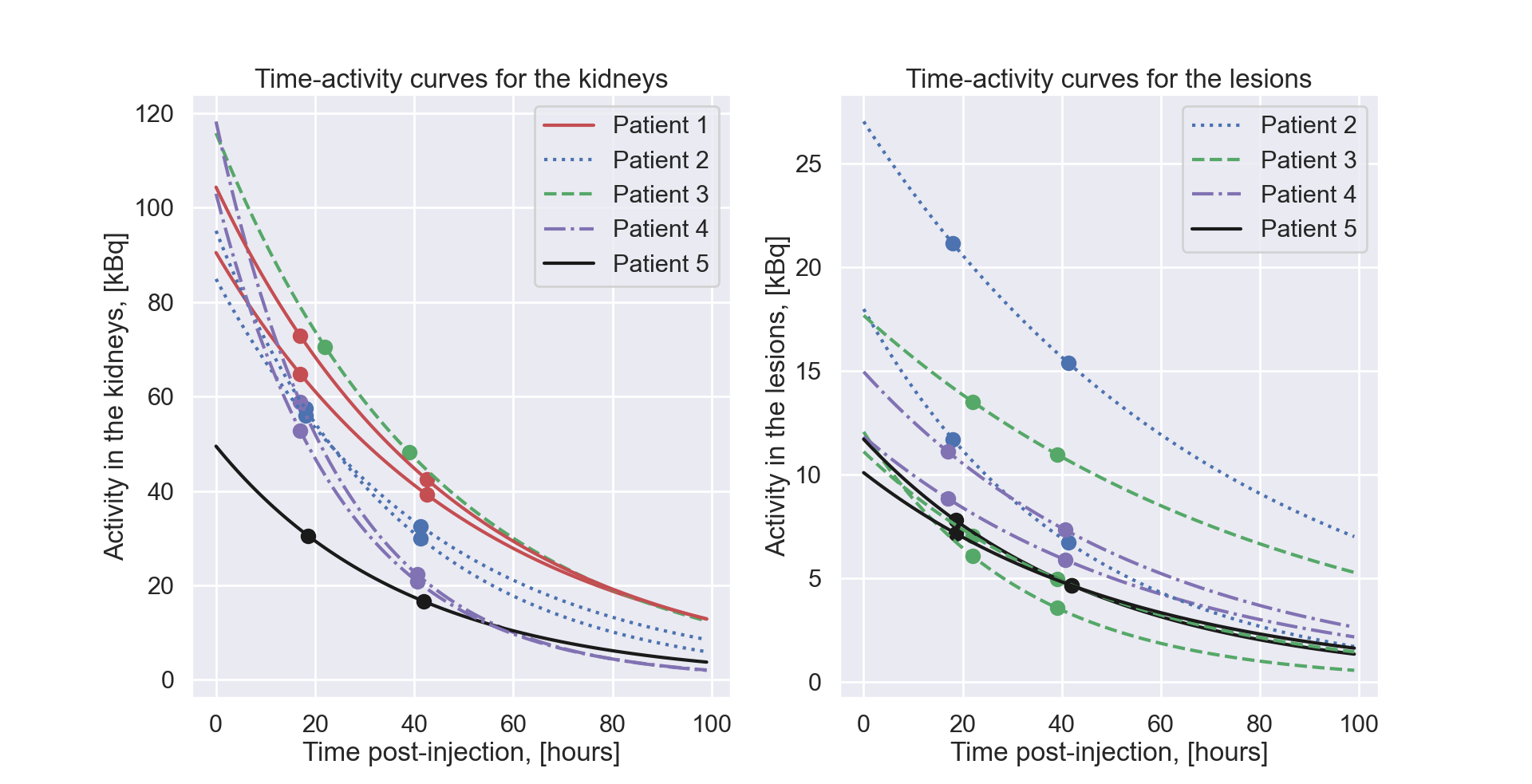


Figure A1: Time-activity curves for the analyzed kidneys (left) and lesions (right) for all patients. The two data points (shown with dots) used to generate the mono-exponential fits are also shown.
